# Volume Phase Transitions of Slide-Ring Gels

**DOI:** 10.3390/polym8060217

**Published:** 2016-06-03

**Authors:** Akinori Bando, Rumiko Kasahara, Kentaro Kayashima, Yasushi Okumura, Kazuaki Kato, Yasuhiro Sakai, Hideaki Yokoyama, Yuya Shinohara, Yoshiyuki Amemiya, Kohzo Ito

**Affiliations:** Graduate School of Frontier Sciences, The University of Tokyo, 5-1-5 Kashiwanoha, Kashiwa, Chiba 277-8561, Japan; kasahara@molle.k.u-tokyo.ac.jp (R.K.); kayashima@molle.k.u-tokyo.ac.jp (K.Kay.); okumura@cm.kyushu-u.ac.jp (Y.O.); kato@molle.k.u-tokyo.ac.jp (K.Kat.); sakai@molle.k.u-tokyo.ac.jp (Y.Sa.); yokoyama@molle.k.u-tokyo.ac.jp (H.Y.); yuya@k.u-tokyo.ac.jp (Y.Sh.); amemiya@k.u-tokyo.ac.jp (Y.A.)

**Keywords:** slide-ring gel, polyrotaxane, volume phase transition, small-angle X-ray scattering

## Abstract

The volume phase transition of slide-ring gels with freely-movable cross-linking junctions was investigated. Ionic chemical gels with fixed cross-linking junctions undergo volume phase transitions when they have higher than the critical degree of ionization. However, the experimentally-observed critical ionization value for slide-ring gels is much higher than theoretical values for chemical gels. This difference indicates that the volume phase transition is significantly suppressed in slide-ring gels. The mesoscale structure at various swollen or shrunken states was also investigated by small angle X-ray scattering. Changes in the scattering patterns with shrinking slide-ring gels suggest microphase separation due to the sliding of cyclic molecules threaded along the axis of the polymer chains, which may suppress the volume phase transition. In addition, slide-ring gels absorbed/desorbed greater than equilibrium volumes in the shrinking/swelling processes and showed slow dynamics; these observations are also related to their sliding properties.

## 1. Introduction

**Figure 1:**
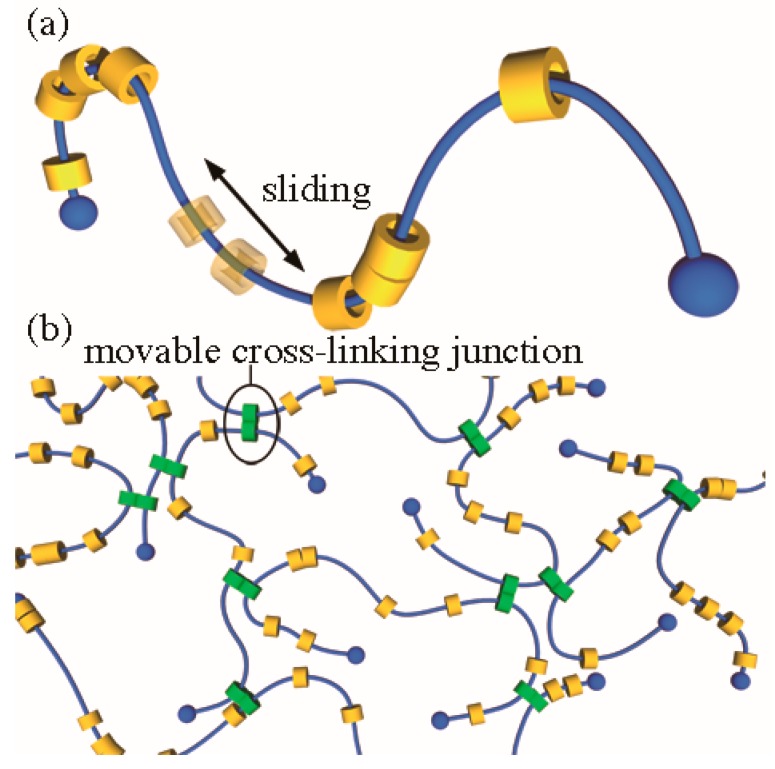
Schematic illustrations of (**a**) PR and (**b**) SR gels.

A slide-ring (SR) gel [1] was obtained by cross-linking cyclic molecules of different polyrotaxanes (PR) [2,3] that have cyclic molecules threaded along the axis of a polymer chain capped by bulky end groups (Figure 1). Having movable cross-linking junctions, SR gels have novel mechanical and swelling properties, such as high extensibility (over 20 times in length), huge swellability (up to 24,000 times by weight), and extremely low Young’s moduli [1,4,5,6,7,8]. The cross-linking junctions in SR gels can slide along the axis of the polymer chains unlike the cross-linking junctions in chemical gels. Therefore, internal stress is reduced by exchange between highly-extended and shrunken chains through cross-linking junctions, the so-called “spulley effect” [1]. Another crucial and unique feature of SR gels is the entropy of rings [9,10,11,12]. SR gels have small Young’s moduli that are not proportional to the cross-linking density and are much lower than those of chemical gels with the same density. This arises from the difference in the molecular mechanism of the entropic elasticity; that is, while the conformational entropy is mainly responsible for the elasticity of chemical gels, the mechanical properties of SR gels are inherently governed by the distribution entropy of free rings in the SR gel as well as the conformational entropy of the backbone polymer. The pulley effect and the entropy of rings are the causes of the various novel physical properties of SR gels, including anomalous stretching-induced swelling behavior [13,14], peculiar nonlinear elasticity [15,16], dynamic transition between rubber, sliding states [9,10,17], and pressure-induced nonlinear penetration flow properties of gel membranes [18]. Due to their novel physical properties and peculiar behaviors, SR gels are expected to be new functional soft materials.

The swelling-shrinking behavior of polymer gels is important for industrial applications. Their peculiar physical properties, such as volume phase transition, have attracted the interest of many scientists. Volume phase transition is the phenomenon of the drastic swelling or shrinking of gels on a small change in the external environment, such as the chemical composition of the solvent mixture [19,20,21], temperature [22,23,24], pH [20], salt concentration [25], electric field [26], and irradiation by ultraviolet [27] or visible light [28]. It has been experimentally and theoretically established that discontinuous transitions occur in chemical gels when they have a greater degree of ionization than a critical value [20,24]. This behavior is well described by the Flory–Huggins–Tanaka theory [20,29], in which the effect of mixing, the entropy of counterions, and the rubber elasticity are all considered. In chemical gels, the network strand length between cross-linking junctions is constant during deformation unless the cross-linking junctions are broken by large deformations. On the other hand, the network strand length can easily change with deformation in the SR gel because the SR gel has movable cross-linking junctions. Therefore, the rubber elasticity of SR gels is quite different from that of chemical gels and the swelling-shrinking behavior of the SR gel is also expected to differ from those of chemical gels.

In this paper, we describe our systematic investigation of the swelling-shrinking behavior of weakly or strongly ionized SR gels with different solvents; furthermore, we describe the mesoscale structures observed in swollen or shrunken states by small angle X-ray scattering (SAXS). The SR gels are carboxylated or sulfonated for ionization. We used two solvent mixtures, ethanol-water and acetone-water, and we changed the water content to observe and compare the swelling-shrinking behavior, including the volume phase transition, between chemical and SR gels. Interestingly, SR gels show a large overshoot in the swelling or shrinking process. We also measured the SAXS profiles of SR gels at different solvent/water contents to investigate the nanostructure of the SR gels. 

## 2. Materials and Methods

### 2.1. Materials

PR consisting of α-cyclodextrin (CD) and poly(ethylene glycol) (PEG, *M*_w_ = 35,000) end-capped with adamantane was purchased from Advanced Softmaterials Inc. (Tokyo, Japan). The inclusion ratios [30], which are the filling ratio of CDs to a full filling state, ranged from 25% to 28%. All chemicals and solvents were purchased from Sigma-Aldrich (Tokyo, Japan) or Wako Pure Chemical Industries, Ltd. (Osaka, Japan) and used without further purification.

### 2.2. Preparation of Carboxylated SR Gels

**Scheme 1:**
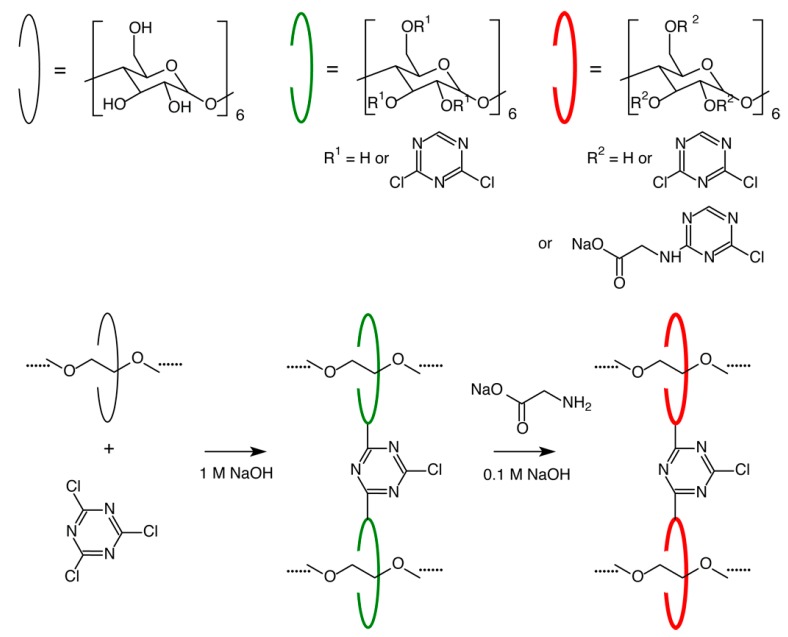
The cross-linking reaction of CDs in PRs with cyanuric chloride and the subsequent introduction of carboxylic acid groups to the SR gels. PEG is omitted here for simplification.

Cross-linking of PR and introduction of the carboxyl group into SR gels were performed as shown in Scheme 1. PR (100 mg) was dissolved in 1 M NaOH (11 mL) in an ice bath. Cyanuric chloride (105 mg), dissolved in 1 M NaOH (1.5 mL), was added to the solution to initiate the cross-linking reaction. Four SR gels were produced by using different cross-linking times, 1.75–3 h, at room temperature. The gels were formed in a glass capillary of 0.14 mm inner diameter, followed by immersion for 2 h in 0.1 M NaOH (90 mL) in which glycine (10 g) was dissolved to introduce carboxylic acid functional groups into the SR gels. Finally, the gels were immersed in 0.1 M NaCl, followed by pure water to remove residual chemicals. The number of carboxyl groups per CD was estimated to be 1.0–1.6 by neutralization titration, as shown in Supplementary Materials. These swollen gels were used for swelling-shrinking experiments. For SAXS experiments, gels were prepared separately as below. Pre-gel solutions containing cyanuric chloride were prepared as described above. The solution was poured into a mold having gaps of 0.5 mm thickness and gelled for 2 h. Obtained gels were immersed in the glycine-NaOH solution, 0.1 M NaCl, and pure water to remove residual contaminants. The prepared gels were immersed in an acetone-water mixture for a week to be at equilibrium after three exchanges of the solvent mixture. The degree of swelling was measured from the thickness of the gel at each acetone volume fraction.

### 2.3. Preparation of Sulfonated SR Gels

**Scheme 2:**
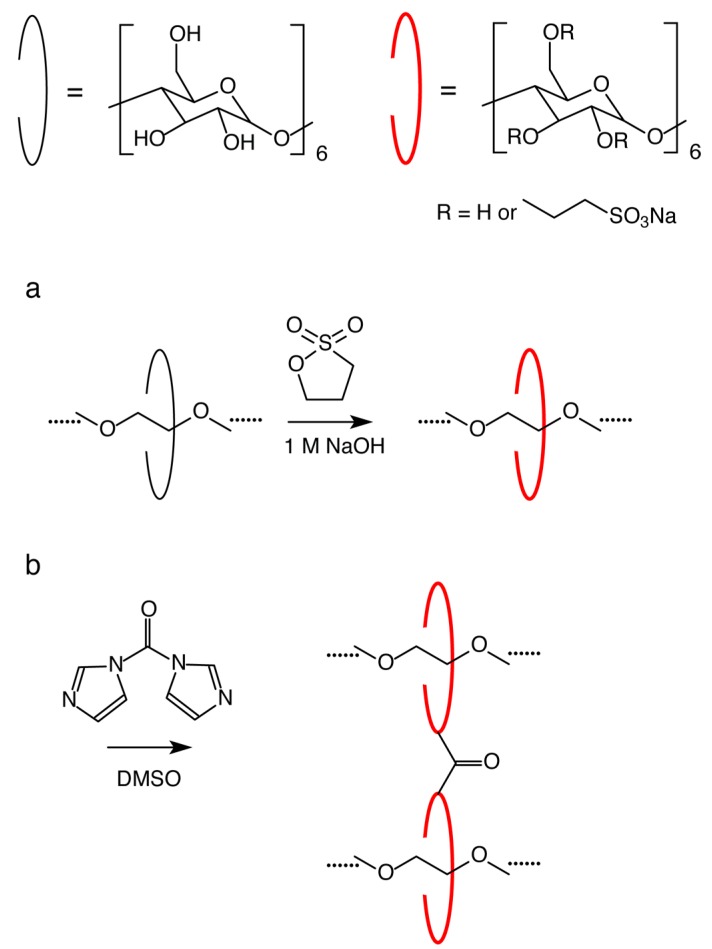
(**a**) Substitution of sulfo groups into polyrotaxane; and (**b**) cross-linking reaction of sulfonated polyrotaxane.

Sulfonated PR was synthesized using ring-opening reactions of 1,3-propane sultone and CD in PR, as shown in Scheme 2a. We prepared four kinds of sulfonated PRs with different numbers, *i,* of sulfo groups per CD, which were estimated by ^1^H NMR: *i* = 0.67, 1.5, 2.8, and 3.4.

Five SR gel samples were prepared with the four sulfonated PRs and one unmodified PR. We used 1,1′-carbonyldiimidazole (CDI) as the cross-linking agent, as shown in Scheme 2b, to estimate the number of cross-linked CDs per PR by Fourier-transform infrared (FT-IR) spectroscopy, as shown in Figures S1–S3. Each PR (45 mg) and CDI (50 mg) was dissolved in dehydrated dimethyl sulfoxide (DMSO, 0.2 mL), and the solutions were then mixed together to initiate a cross-linking reaction. For all samples, the PRs were gelled at 50 °C in a glass capillary of 0.14 mm inner diameter for four days. After gelation, the gels were removed from the capillary and immersed in saturated NaCl aqueous solution (for highly swollen gels only) and then pure water to wash away residual chemicals.

### 2.4. Measurements: Swelling-Shrinking Experiments

The equilibrium and dynamic swelling studies were carried out at room temperature. We measured the dependency of the degree of swelling of the prepared gels on the ethanol or acetone volume fractions. The prepared gels were immersed in ethanol-water or acetone-water mixtures for equilibrium and dynamic swelling experiments. For the dynamic swelling experiment, a 0.1 M NaCl aqueous solution was used as solvent. The gels and surrounding solvent were sealed in glass cells in which solvent can be exchanged to change the water volume fraction. The gels were initially immersed in a high volume fraction of water, and the water fraction was decreased stepwise to observe the shrinking process; water content was then increased to observe the swelling behavior (solubility of PR is shown in Figure S4). The diameters of the gels were measured by optical microscopy at each water volume fraction. The diameters of the gels in equilibrium gave us the degree of swelling as a function of ethanol or acetone volume fraction. The time dependence of the diameters after the exchange of the solvent was also measured, yielding the degree of swelling as a function of time.

### 2.5. SAXS Experiments

SAXS experiments were performed at the BL-15A, Photon Factory (KEK, Tsukuba, Japan) [31]. The X-ray wavelength, λ, was 0.15 nm. An X-ray CCD detector (C4880–50–26, Hamamatsu Photonics, Hamamatsu, Japan) coupled with a 150 mm diameter X-ray image intensifier [32] was used. The exposure times were changed from 0.2 to 1 s, depending on the sample thickness. The sample-to-detector distance was 2.1 m. Therefore, the SAXS patterns were measured for 0.09 nm^−1^ < *q* < 1.4 nm^−1^, where *q* = 4π(sinθ)/λ is the magnitude of the scattering vector and 2θ is the scattering angle.

The nanoscale structures in carboxylated SR gels in acetone-water mixtures of various acetone volume fractions were measured by SAXS. The acetone volume fractions varied from 0.50 to 0.80 at a step of 0.02. Each gel was set in a columnar hole of an aluminum spacer, which was sealed with Kapton films to prevent drying of the gel. The scattering profiles of acetone-water mixtures of almost the same thickness as the corresponding gels were measured to subtract the scattering from the solvent. The subtracted scattering profiles were then normalized by exposure time, sample thickness, and the volume fraction of the polymer network.

## 3. Results

### 3.1. Swelling-Shrinking Behaviors of Carboxylated SR Gels

**Figure 2:**
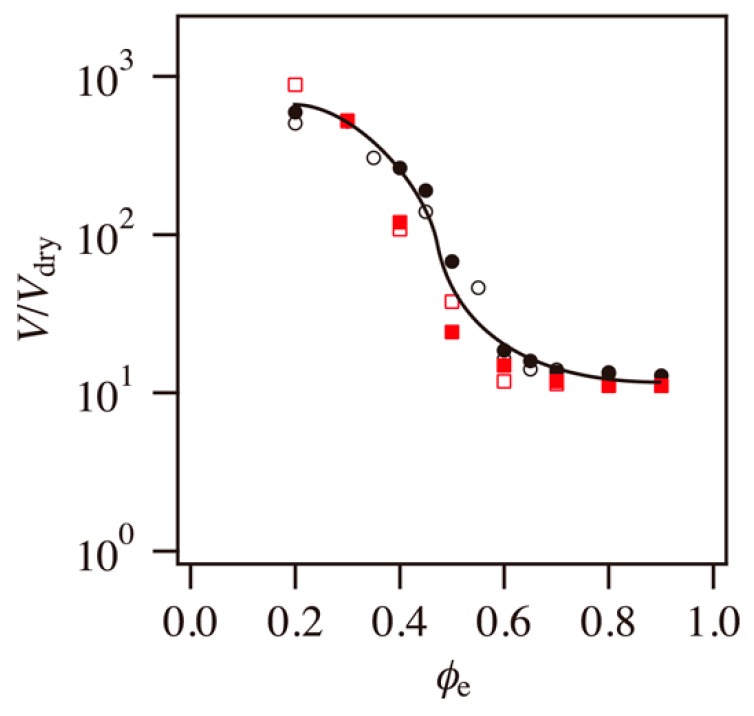
The degree of swelling *V*/*V*_dry_ of the carboxylated SR gels with cross-linking times of 1.45 h (circle) and 2 h (square) as a function of ethanol volume fraction, $\phi_{\text{e}}$. Filled and open symbols denote shrinking and swelling processes, respectively. The solid line is an eye guide.

Figure 2 shows the dependence of the degree of swelling for two carboxylated SR gels on the ethanol volume fraction in a mixture of ethanol and water. The maximum degree of swelling was nearly one thousand times by dry weight. Such a large volume change suggests that the carboxylic acid functionalities were certainly introduced into the SR gels. The volume of the SR gel was changed 100 times continuously without hysteresis in the solvents, between low and high ethanol fractions. Generally, ionized chemical gels that show such large volume changes show discontinuous volume phase transitions. Consequently, it appears that volume phase transitions do not occur easily in these SR gels. In addition, the swelling and shrinking behaviors of the SR gels are almost independent of cross-linking time in ethanol and water mixtures.

**Figure 3:**
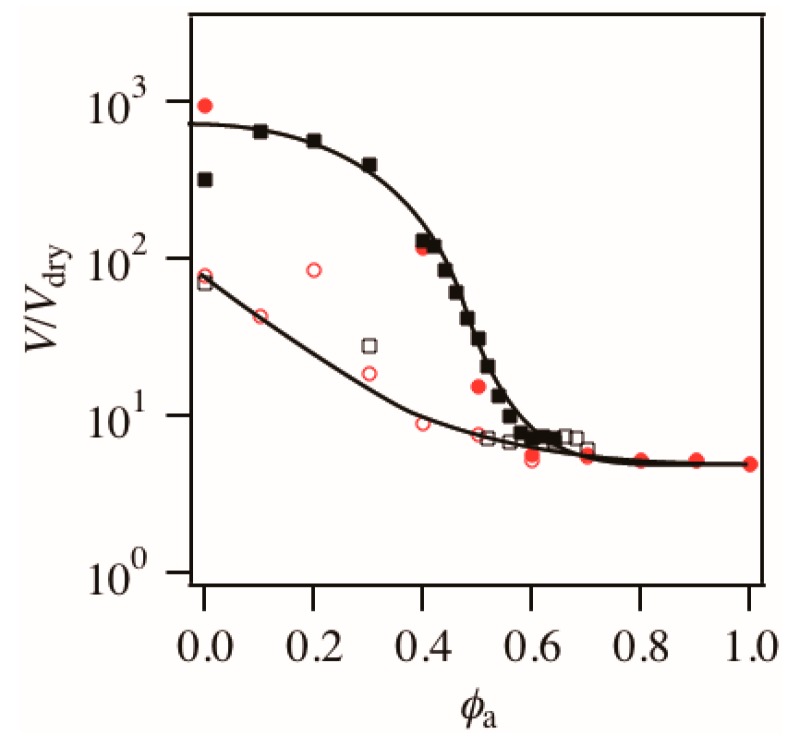
The degree of swelling *V*/*V*_dry_ of the carboxylated SR gels with cross-linking times of 2.7 h (circle) and 3 h (square) as a function of acetone volume fraction, $\phi_{\text{a}}$. Filled and open symbols denote shrinking and swelling processes, respectively. The solid lines are eye guides.

The dependence of the degree of swelling of the carboxylated SR gels on the acetone volume fraction in the mixture of acetone and water also indicate that there was no volume phase transition, and this was also observed in the ethanol-water mixture, while hysteresis was seen only in the acetone-water mixture (Figure 3). Furthermore, the SR gel had lower degree of swelling in its shrunken state in the acetone-water mixture than in the ethanol-water one. These results mean that PR forms more cohesive aggregates in acetone-rich solvents than in ethanol-rich solvents. The swelling and shrinking behaviors of the carboxylated SR gels were almost independent of the cross-linking time in both acetone-water and ethanol-water mixtures.

### 3.2. Swelling-Shrinking Behaviors of Sulfonated SR Gels 

**Figure 4:**
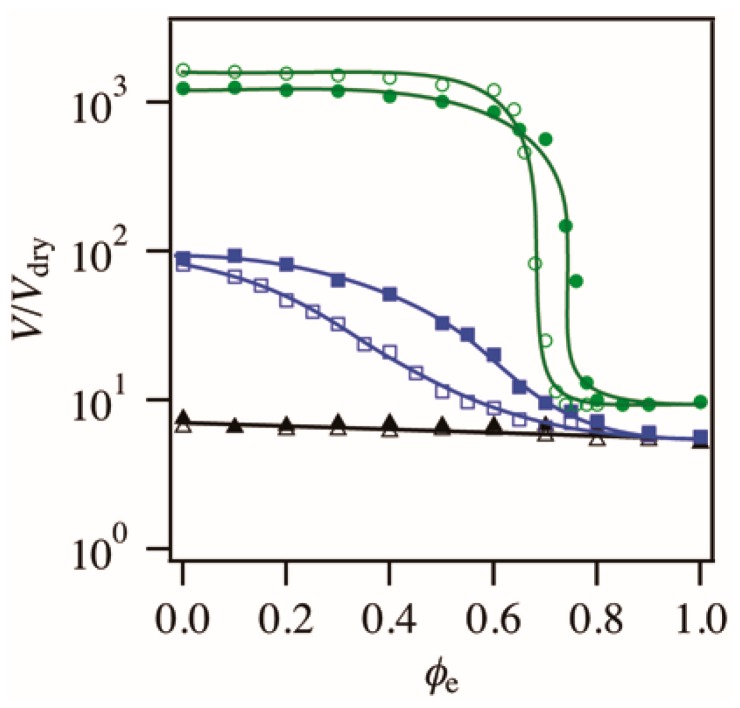
The degree of swelling *V*/*V*_dry_ of sulfonated SR gels in the lower ionization region as a function of ethanol volume fraction, $\phi_{\text{e}}$, in the ethanol-water mixture. The average numbers of sulfo groups per CD were 0 (triangle), 0.67 (square), and 1.5 (circle). Filled and open symbols denote the shrinking and swelling processes, respectively. Solid lines are eye guides.

**Figure 5:**
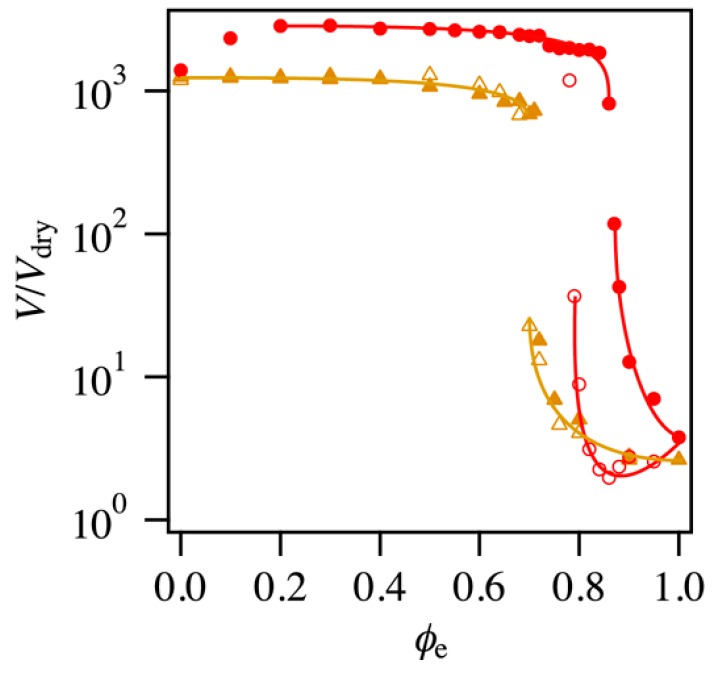
The degree of swelling *V*/*V*_dry_ of sulfonated SR gels in the higher ionization region as a function of ethanol volume fraction $\phi_{\text{e}}$ in the ethanol-water mixture. The average numbers of sulfo groups per CD were found to be 2.8 (triangle) and 3.4 (circle). Filled and open symbols denote the shrinking and swelling processes, respectively. Solid lines are eye guides.

**Figure 6:**
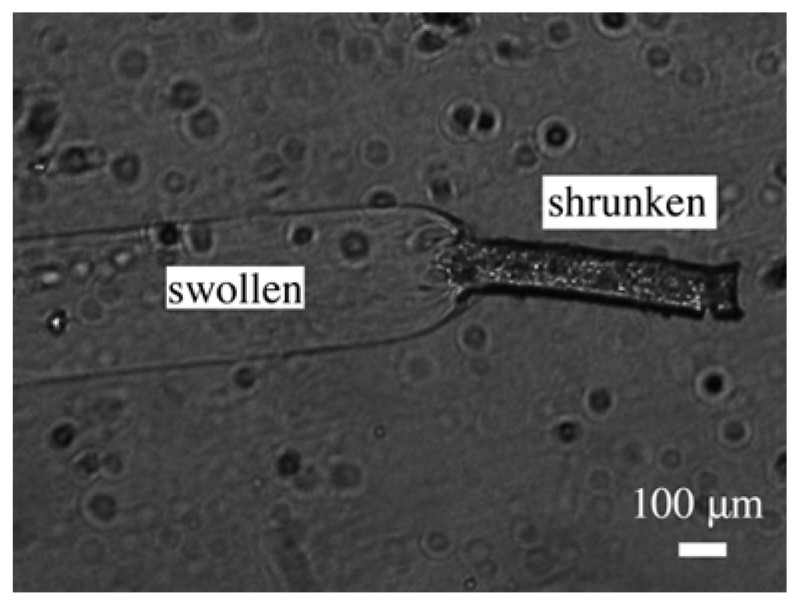
Coexistence of swollen and shrunken phases in the sulfonated SR gels at *i* = 2.8 and $\phi_{\text{e}}$ = 0.71. This coexistence state lasted for several days.

Next, we measured the swelling and shrinking behaviors of the sulfonated SR gels, which have various substitution ratio, in an ethanol-water mixture. A neutral SR gel did not depend on the ethanol volume fraction, because both ethanol and water were poor solvents of unmodified PR (Figure 4). The dependence of the degree of swelling for two sulfonated SR gels at lower *i* (0.67 and 1.5) on the ethanol volume fraction indicated that the SR gels swelled or shrank continuously in an unionized or small degree of ionization region (Figure 4); however, a large and drastic volume change was observed at *i* = 1.5. In contrast, the SR gels in the higher ionization region show discontinuous volume changes at higher values of *i*, *i.e.*, 2.8 and 3.4, as shown in Figure 5. Figure 6 shows the picture of the SR gel at *i* = 2.8 and $\phi_{\text{e}}$ = 0.71 in the shrinking process. The coexistence of swollen and shrunken phases was clearly observed, which indicates a definite evidence of the volume phase transition. Shrinkage of the SR gels at *i* = 2.8 took several days to shrink uniformly and completely.

### 3.3. SAXS Profiles of Carboxylated SR Gels in Acetone-Water Mixture. 

**Figure 7:**
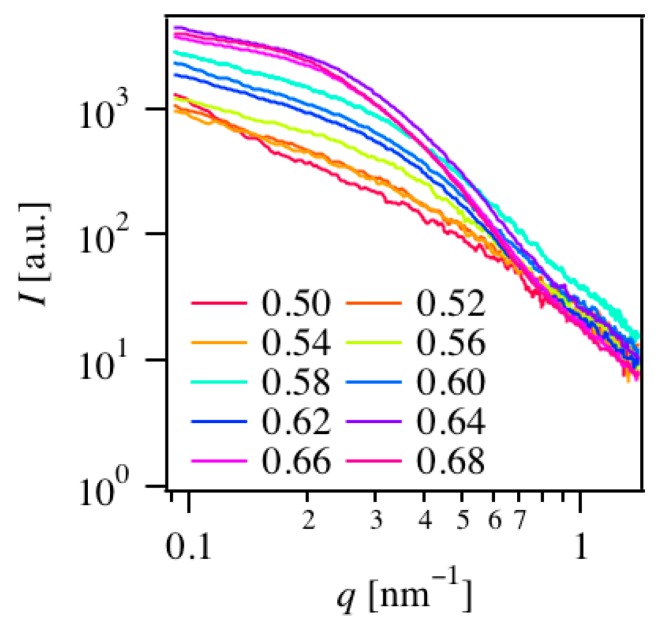
Circularly-averaged SAXS intensity profiles of the carboxylated SR gels at $\phi_{\text{a}}$ = 0.50–0.68.

**Figure 8:**
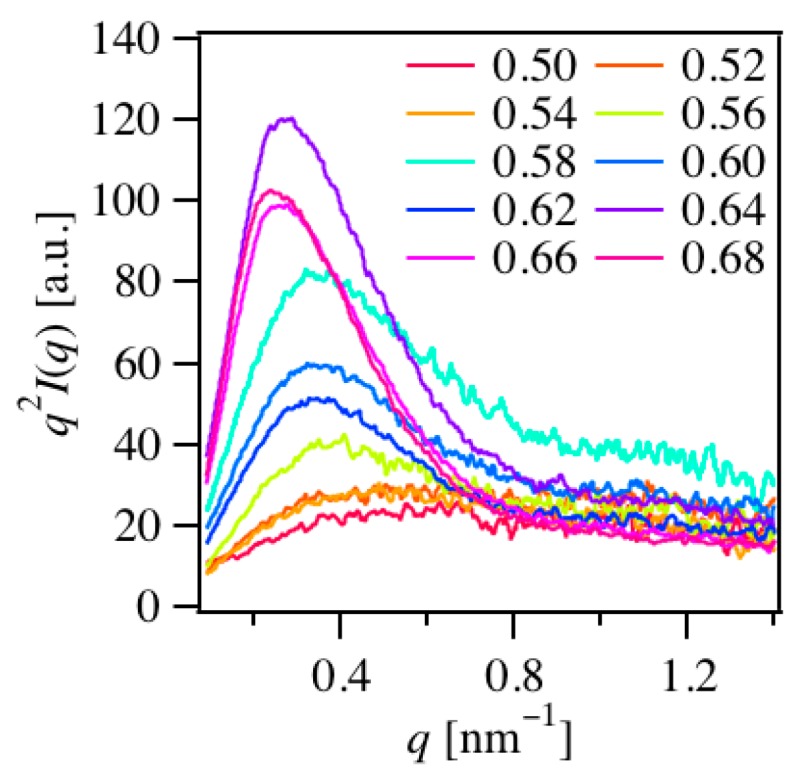
Kratky plots of the carboxylated SR gels at $\phi_{\text{a}}$ = 0.50–0.68.

**Figure 9:**
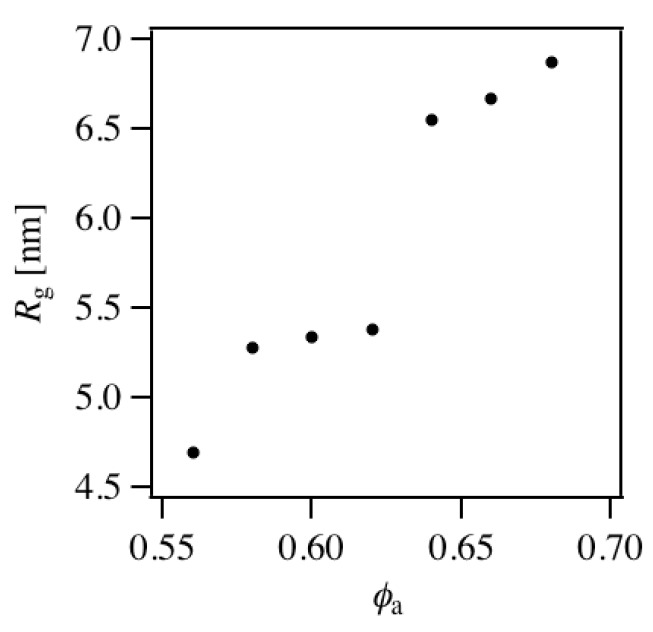
The radius *R*_g_ of gyration of the aggregated domain as a function of $\phi_{\text{a}}$.

Figure 7 shows the circularly averaged SAXS intensity profiles of the carboxylated SR gels at $\phi_{\text{a}}$ = 0.50–0.68. The SAXS intensity profiles of the SR gels in the lower acetone fractions of $\phi_{\text{a}}$ = 0.52 and 0.54 were well fitted by the Ornstein-Zernike function:

(1) $$I_{1}(q)=\frac{A}{1+\xi^{2}q^{2}}$$

where *A* is the scattering intensity at *q* = 0 and ξ the length of correlation blob (Figure S5 in the Supplementary Material). This suggests that polymer chains in $\phi_{\text{a}}$ ≤ 0.54 behave as Gaussian chains. The profiles of the SR gels at higher acetone fractions ($\phi_{\text{a}}$ ≥ 0.56) do not fit the Ornstein-Zernike function well. Therefore, we used a Kratky plot [33] *q*^2^*I*(*q*) *vs*. *q*, as shown in Figure 8. Obvious peaks appear in the Kratky plot for the range 0.56 ≤ $\phi_{\text{a}}$ ≤ 0.68, indicating the existence of some aggregated domains in the SR gel. These scattering profiles are fitted well by the Guinier function:

(2) $$I_{2}(q)=I_{g}\exp\left(-\frac{R_{g}^{2}q^{2}}{3}\right)$$

where *I*_g_ is the scattering intensity at *q* = 0 and *R*_g_ the radius of gyration of the domain (Figures S6 and S7 in Supporting Information). Figure 9 shows the dependence of *R*_g_ in the SR gels on $\phi_{\text{a}}$ in 0.56 ≤ $\phi_{\text{a}}$ ≤ 0.68, indicating the growth of the domain with increasing acetone fraction.

**Figure 10:**
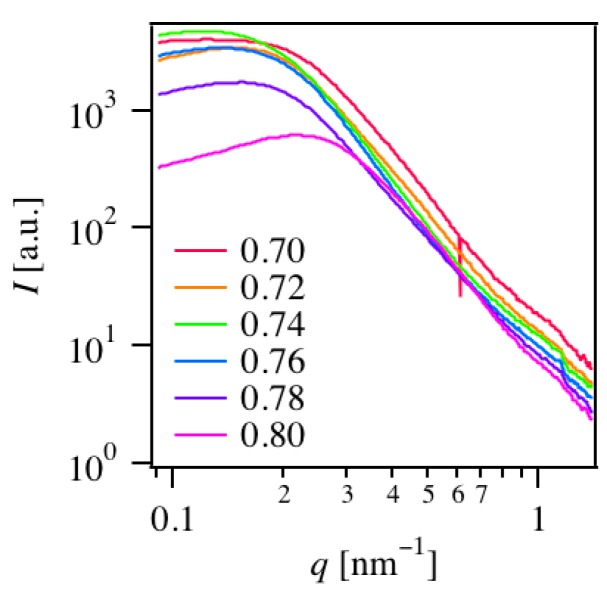
Circularly-averaged SAXS intensity profiles of the carboxylated SR gels at $\phi_{\text{a}}$ = 0.70–0.80.

**Figure 11:**
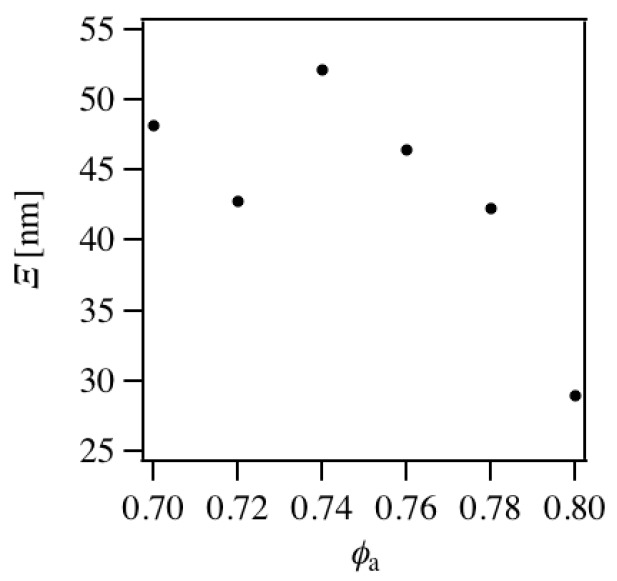
The distance Ξ between aggregated domains as a function of $\phi_{\text{a}}$.

On the other hand, peaks are also observed in the scattering profiles of the SR gels at $\phi_{\text{a}}$ = 0.70–0.80, as shown in Figure 10. The profiles can be fitted with the form reflecting the correlation between these aggregations:

(3) $$I_{3}(q)=B\exp\left[-C{\left(q-q_{c}\right)}^{2}\right]$$

where *q*_c_ = 2π/Ξ and Ξ represents the distance between neighboring domains (Figure S8 in the Supporting Information). Figure 11 shows the dependence of Ξ in the SR gels on $\phi_{\text{a}}$ in 0.70 ≤ $\phi_{\text{a}}$ ≤ 0.80, indicating that Ξ initially tends to increase slightly and then decrease with increasing $\phi_{\text{a}}$.

### 3.4. Dynamic Swelling-Shrinking Behaviors of SR Gels. 

**Figure 12:**
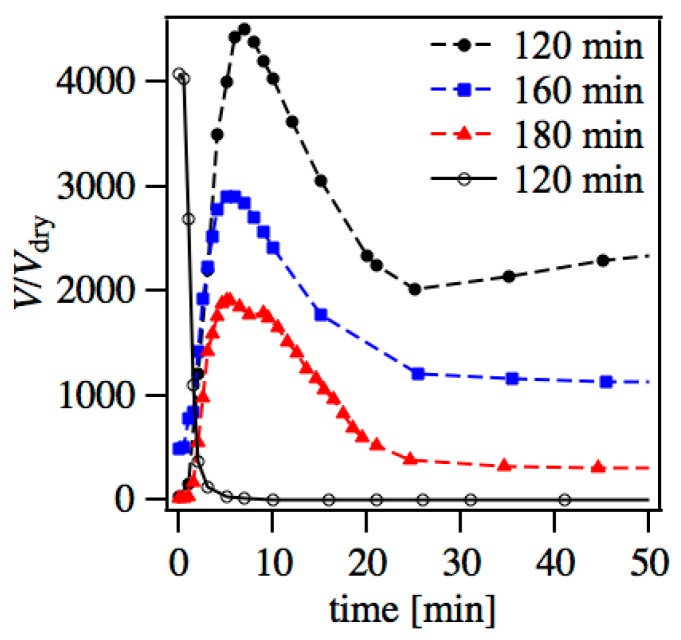
The degree of swelling *V*/*V*_dry_ of the carboxylated SR gels as a function of time when solvent was exchanged from 0.1 M NaCl aqueous solution to water (dashed line) and from water to 0.1 M NaCl aqueous solution (solid line). Cross-linking times are 120 min (circle), 160 min (square), and 180 min (triangle), respectively.

Figure 12 shows the degree of swelling of the carboxylated SR gels as a function of time when solvent was exchanged from 0.1 M NaCl aqueous solution to water and, back again, from water to 0.1 M NaCl aqueous solution. The carboxylated SR gels exhibit a solvent uptake greater than the equilibrium value (overshoot) during the swelling process, but not in the shrinking process. The overshoot was observed even in the shrinking process in acetone-water or ethanol-water mixtures, although it was not seen in the swelling process, as shown in Figure S9. Immediately after solvent is exchanged from $\phi_{\text{a}}$ = 0.20 to $\phi_{\text{a}}$ = 0.30, or from $\phi_{\text{a}}$ = 0.30 to $\phi_{\text{a}}$ = 0.40, the volume of the SR gel expands rapidly, and then shrank gradually and, finally, returned to the equilibrium state in several days. 

## 4. Discussion

As shown in Figure 2 and Figure 3, the carboxylated SR gels did not show discontinuous transitions. Carboxylic acid groups do not have a sufficient effect to cause the volume phase transition in SR gels, though the transition was observed in carboxylated chemical gels [24]. This result suggests that volume phase transitions do not easily occur in SR gels. In sulfonated SR gels, a discontinuous volume phase transition is clearly observed when *i* ≥ 2.8, as shown in Figure 5. On the other hand, SR gels swelled or shrank continuously when *i* ≤ 1.5, although a large and drastic volume change occurred when *i* = 1.5 (Figure 4). This indicates that the critical degree of ionization, *i*_c_, for the volume phase transition is approximately 2 in the SR gel. On the other hand, the critical value of the volume phase transition was evaluated to be *i*_c_ = 0.63 in the chemical gel, obtained from a numerical calculation using Flory-Rehner-Tanaka theory, a value similar to those obtained experimentally [24]. Thus, the experimental values of *i*_c_ in the SR gels are much higher than the theoretical values for the chemical gels. This means that the volume phase transition is significantly suppressed in SR gels.

We have recently proposed a new theoretical model of the swelling and shrinking behavior in SR gels. In this model, the sliding of effective strands to dangling ones through cross-linking junctions is considered [34]. From the model, the critical degree of ionization of SR gels used in this experiment was estimated to be *i*_c_ = 0.70, slightly higher than the theoretical value obtained from Flory-Rehner-Tanaka theory (= 0.63) for a chemical gel under the same conditions. This supports the results that the volume phase transition is suppressed in SR gels. However, the theoretical value obtained from the model of SR gels is still lower than the experimental one (≈2). We discuss this discrepancy between the theory and experiment from the results of SAXS.

From the SAXS profiles, mesoscale structures of the SR gel can be classified into three regions: the SAXS patterns were fitted well by the Ornstein-Zernike function in region I ($\phi_{\text{a}}$ = 0.50–0.54), the Guinier function in region II ($\phi_{\text{a}}$ = 0.56–0.68), and by Equation (3) in region III ($\phi_{\text{a}}$ = 0.70–0.80). In region I, described by the Ornstein-Zernike function, PRs behave as Gaussian chains and CDs are dispersed sparsely.

**Figure 13:**
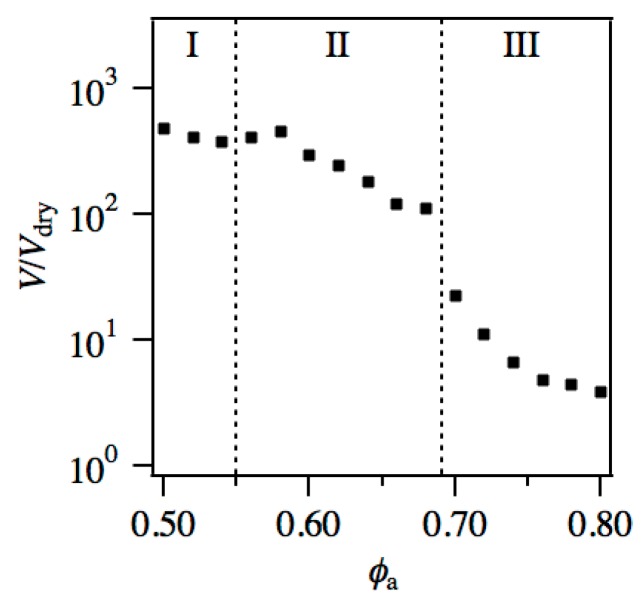
The degree of swelling *V*/*V*_dry_ of the carboxylated SR gels as a function of $\phi_{\text{a}}$ in shrinking process. From the SAXS profiles, the mesoscale structure of the SR gel can be classified into three regions (I–III).

In the other regions at $\phi_{\text{a}}$ ≥ 0.56, the SR gels show different profiles suggesting formation of some aggregates. Shinohara *et al*. reported that, using SAXS, aggregates of CDs were observed in the SR gel in poor solvents [35]. Hence, the domain in the SR gel in regions II and III should be assigned to the aggregation of CD molecules. Namely, *R*_g_ in Equation (2) indicates the domain size of the CD aggregation, which increases at $\phi_{\text{a}}$ = 0.58 to 0.68, as shown in Figure 9. Figure 13 shows the degree of swelling of the SR gel as a function of acetone volume fraction. Between $\phi_{\text{a}}$ = 0.54 to 0.60, the SR gel is not shrinking, in spite of the formation of CD aggregates. This suggests that the CD aggregates are enhanced by the sliding of CDs and that the backbone polymer does not collapse, maintaining the network especially at lower values of $\phi_{\text{a}}$.

The structure of the aggregates and the volume of the SR gel change simultaneously at $\phi_{\text{a}}$ = 0.70. A peak was observed in the SAXS profiles at $\phi_{\text{a}}$ values between 0.70 and 0.80. This indicates that the distance between the aggregated domains become uniform, averaging around 50 nm. The distance increased from $\phi_{\text{a}}$ = 0.70 to 0.74 and then decreased from $\phi_{\text{a}}$ = 0.74 to 0.80. The change in distance arises from the competition between the growth of aggregations and the shrinkage of the gel; the former enhances the separation between aggregated domains, whereas the latter decreases it.

When *q* is normalized with ξ, we found that the SAXS profiles at $\phi_{\text{a}}$ = 0.56–0.68 in region II during the shrinking process are on two master curves related by a vertical shift, as shown in Figure S10. Therefore, the structure at $\phi_{\text{a}}$ = 0.56–0.62 is considered to be slightly different from that at $\phi_{\text{a}}$ = 0.64–0.68. The SAXS profiles in the low-*q* region are well fitted to the Guinier function at $\phi_{\text{a}}$ = 0.64–0.68, as shown in Figure S7. However, a discrepancy is seen in the low-*q* region at $\phi_{\text{a}}$ = 0.56–0.62, as shown in Figure S6. Furthermore, a drastic increase in the size of aggregates is observed at $\phi_{\text{a}}$ = 0.64, as shown in Figure 9. This suggests that the growth of the aggregates narrows the size distribution with clearer boundaries between the CD aggregations and the swollen PEG chains around $\phi_{\text{a}}$ = 0.64 as the acetone volume fraction increases from $\phi_{\text{a}}$ = 0.56 to 0.68.

**Figure 14:**
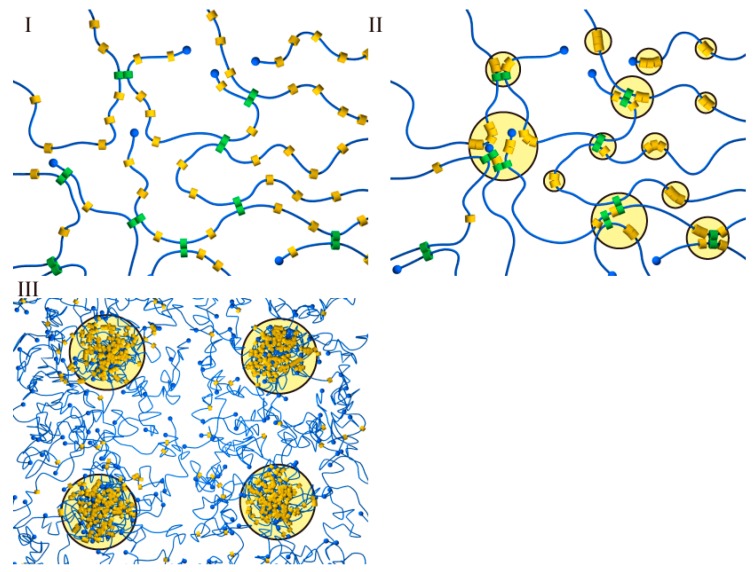
Schematic illustrations of the mesoscale structure of carboxylated SR gels in acetone/water mixtures. In the region I, polymer chains and CDs are distributed sparsely. As the acetone fraction increases, the carboxylated CD in the SR gels should lose the charge and form aggregation, and chains slide to swollen domain in region II. The aggregated and swollen domains are more clearly separated like a micro phase separation, yielding a peak in SAXS pattern in region III.

The schematic illustrations of the mesoscale structure in the region I–III are shown in Figure 14. In good solvents, PRs in the SR gel have a Gaussian conformation and CDs are distributed randomly (region I). When acetone volume fraction increases, some CDs slide to form aggregates with slight volume shrinkage and the growth of the aggregations narrows the size distribution of the aggregated domains (region II). Then the separation between the aggregated domains becomes so uniform to yield a peak in the SAXS profile (region III). This can be regarded as a kind of microphase separation, where CD-rich domains are separated from PEG and solvent. 

Now, we discuss the quantitative discrepancy between experimental and theoretical results in the critical degree of ionization for the volume phase transition. The theoretical model of the SR gels assumed that the chain length between cross-linking junctions was homogeneous. In this model, we considered only the sliding of chains between dangling and elastically-effective chains through cross-linking junctions. However, the SAXS profiles clearly indicate that the aggregated domains are formed and grow in the SR gels as the solvent becomes poor. This suggests that the sliding of CDs occurs between the aggregated and swollen domains in the SR gel. This effect has not been considered in the theoretical model of the homogeneous SR gel. 

On the other hand, the chemical gel has cross-linking junctions that cannot slide along the polymer chains. If chains form shrunken and swollen domains in the gel, the gel becomes elastically unfavorable. Consequently, the chemical gels cannot easily form the microphase separation of shrunken and swollen domains of chains. In contrast, the microphase separation in SR gels can be stabilized by the chains sliding through cross-linking junctions from aggregated domains to swollen domains. Therefore, the sliding of chains and CDs tends to suppress the drastic volume change of the SR gels. In this way, the quantitative discrepancies between the experimental and theoretical results can be explained by the existence of the microphase separation structure and underestimation of the sliding of CDs or chains through cross-linking junctions.

This picture is also related to the large overshoot phenomena of SR gels in the swelling process. The dynamics of the swelling and shrinking behaviors in the chemical gels is mainly dominated by the solvent flow through the network with the chain friction [20]. However, the sliding of CDs and chains in the SR gel yields a changeable network size, as shown in the pressure-induced nonlinear penetration flow properties of SR gel membranes [18]. This may explain both the overshoot in the swelling and shrinking processes and the slow dynamics of the SR gels; that is, the solvent flow expands the network size via the sliding cross-linking junctions, leading to temporary large-scale swelling followed by shrinkage. The strong coupling between the solvent flow and changeable network size slows down the dynamics of the SR gel. Consequently, the sliding of CDs and chains affects the swelling and shrinking behaviors of the SR gel, including the volume phase transition. 

## 5. Conclusions

We investigated the dependence of the degree of swelling in weakly and strongly ionized SR gels on the chemical composition of solvent mixtures (ethanol-water or acetone-water). We also measured the mesoscale structure of the SR gels at each degree of swelling by SAXS. The weakly ionized SR gels did not show discontinuous volume changes, while the strongly ionized SR gels showed a volume phase transition, but had a much higher critical degree of ionization than the theoretical value determined by the Flory–Rehner–Tanaka model. These results indicate that the volume phase transition is significantly suppressed in the SR gel. The theoretical model of the SR gels showed the suppression of the volume phase transition in the SR gels qualitatively, though the evaluated critical value was still lower than the experimental one. Using SAXS measurements, we also found various mesoscale structures of the SR gels on their gradual shrinkage. These were classified into three regions. Polymer chains and CDs were distributed randomly in region I. When the acetone volume fraction increased, CDs slid along the polymer chains to form aggregates with slight shrinkage in region II. With increasing acetone volume fraction, the aggregated domains grew and the size distribution of the aggregated domains narrowed. In region III, where the SR gel shrank significantly, the distance between the aggregated domains had a similar correlation to that of the microphase separation, decreasing with increasing acetone volume fraction, which was stabilized elastically by the sliding of CDs and chains. The quantitative discrepancy between the theoretical and experimental results concerning the critical values can be explained by the existence of the microphase separation structure and underestimation of the sliding of CDs or chains through cross-linking junctions. This is also related to the overshoot in the swelling or shrinking process and the slow dynamics of the SR gels. Consequently, the sliding of CDs and chains affects the swelling and shrinking behavior of the SR gel significantly including the volume phase transition. 

## Supplementary Materials

Supplementary materials can be found at www.mdpi.com/2073-4360/8/6/217/s1.

## Abbreviations

The following abbreviations are used in this manuscript:

PEG: Poly(ethylene glycol)
CD: Cyclodextrin
SR: Slide-ring
PR: Polyrotaxane
SAXS: Small-angle X-ray scattering
CDI: 1,1′-carbonyldiimidazole
