# Supplementary material for: Volume Phase Transitions of Slide-Ring Gels
Source: Polymers (Basel). 2016 Jun 3;8(6):217. doi: 10.3390/polym8060217 (PMC6432380; doi:10.3390/polym8060217)
Supplement: Supplementary file 1 [file polymers-08-00217-s001.pdf]

Supplementary Materials: Volume Phase Transitions of Slide-Ring Gels

Akinori Bando, Rumiko Kasahara, Kentaro Kayashima, Yasushi Okumura, Kazuaki Kato, Yasuhiro Sakai, Hideaki Yokoyama, Yuya Shinohara, Yoshiyuki Amemiya and Kohzo Ito

1. Estimation of the Number of Carboxylic Groups per CD

The mass molar concentration of counter ions in dried SR gel, ρ, was estimated to be 0.69 mmol/g by neutralization titration. The molar weight of PR in dried SR gel, M_{PR}, is given by

$$M_{PR} = n_{CD}(M_{CD} + M_x \times x + M_i \times y) + M_{PEG} \quad \text{(S1)}$$

where n_{CD} is the number of CDs per PR, M_{CD} the molar weight of CD, M_x the molar weight of cross-linking junctions, x the number of cross-linking junctions per CD, M_i the molar weight of ionization groups per CD, y the number of ionization groups per CD, M_{PEG} the molar weight of PEG. The number of carboxylic acid functional groups per CD was given by

$$y = \frac{\rho M_{PR}}{n_{CD}} \quad \text{(S2)}$$

The number of cross-linking junctions per CD is limited to $x + y < 7.3$ from the amount of cyanuric chloride. The number of carboxylic acid functional groups per CD was estimated to be 1.0–1.6.

2. Estimation of the Number of Cross-Linked CDs per PR

The number of cross-linked CDs per PR, m, was estimated from the peak area of the carbonyl stretching from the FT-IR spectra because the SR gel cross-linked with CDI forms a carbonate ester.

SR gels soaked in pure water were freeze-dried. IR spectra of the dried gels were measured using a Nicolet 4700 FT IR spectrometer (Thermo Electron Co., Waltham, MA, USA) equipped with a diamond attenuated total reflection (ATR) accessory (DurasamplIR II. SensIR Technologies, Tokyo, Japan).

Figure S1 shows the IR spectrum of the dried SR gel and fitting curves. The carbonyl stretching corresponds to the peak at 1750 cm^{-1}. An absorption peak at 2880 and 2920 cm^{-1} are attributed to the methylene stretching of CD and PEG, respectively. The IR spectrum was fitted by Gaussian models with these three peaks and that of water at 1650 cm^{-1}. The number of cross-linked CDs per PR should be related to the peak area ratio of the carbonyl to methylene stretching of CD.

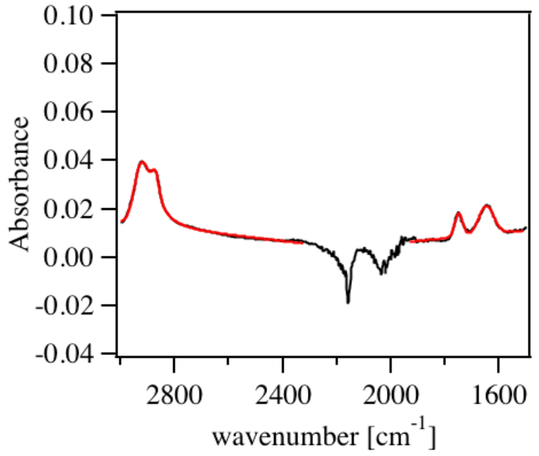

Figure S1. ATR-FTIR spectrum of the dried SR gel (black line) and fitting curves (red line).

The relationship between the number of cross-linked CDs per PR and the peak area ratio was obtained using the relationship between the IR spectrum and SEC profile of cross-linked CD. CD was cross-linked with CDI in DMSO at the same as the above SR gel samples. The cross-linking reaction was quenched with deionized water. The solution was sealed in a dialysis membrane (100–500 Da cutoff) to remove residual chemicals. The resultant solution was then freeze-dried, and the peak area ratio of the carbonyl stretching to the methylene stretching in IR spectrum was measured.

SEC measurements were performed on a GPC KD-802.5 (Shodex, Tokyo, Japan) with DMSO with 10 mM LiBr as eluent at 323 K and a flow rate of 0.5 mL/min using RI detection. Figure S2 shows SEC profiles of CDs cross-linked with various concentrations of CDI. The peak at 32 min corresponds to uncrosslinked CDs. Shoulders or peaks earlier than 32 min correspond to cross-linked CDs. The ratio of cross-linked CDs to all CDs was evaluated from these areas.

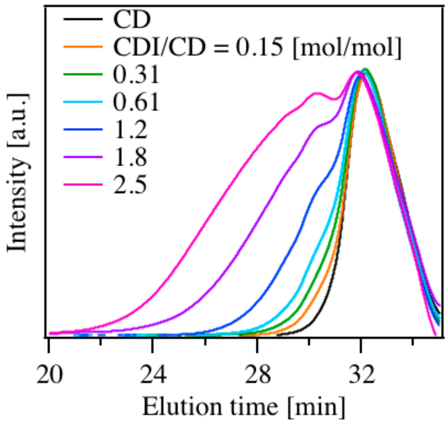

Figure S2. SEC profiles of CDs cross-linked with various concentrations of CDI.

The relationship between the number ratio of cross-linked CDs to all CDs, ϕ_x, and the peak area ratio of the carbonyl stretching and the methylene stretching, $A_{C=O}/A_{C-H}$, is shown in Figure S3. One PR used in this paper has 99 CDs. Then the number of cross-linked CDs per PR is given as $m = 99\phi_x$.

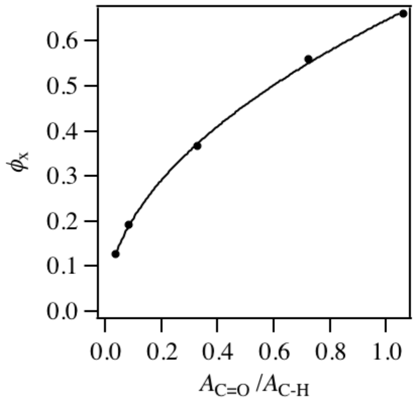

Figure S3. The relation between the ratio of cross-linked CDs to all CDs, ϕ_x, and the peak area ratio of the carbonyl stretching to the methylene stretching, $A_{C=O}/A_{C-H}$.

3. Solubility of PR

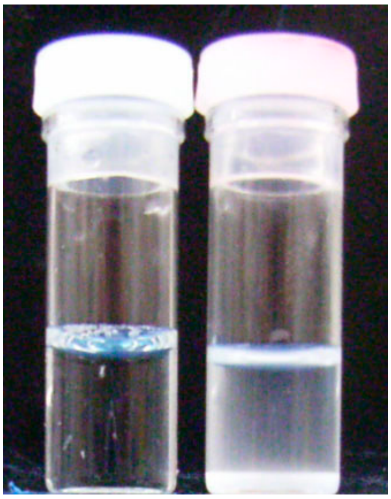

Figure S4. Sulfonated PR was dissolved in pure water (**left**) and precipitated in ethanol (**right**).

4. SAXS Profiles

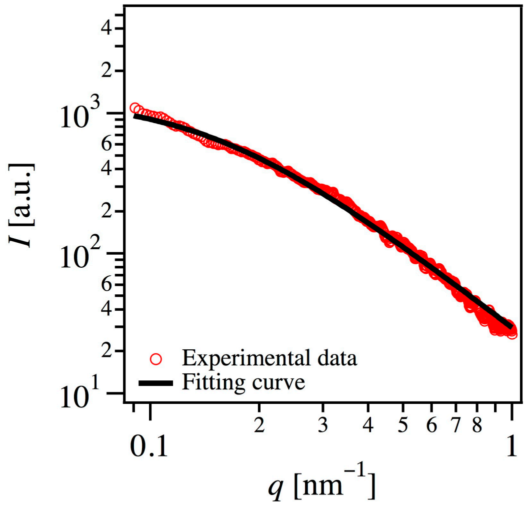

Figure S5. A circularly averaged SAXS intensity profile of the carboxylated SR gels at ϕ_a = 0.52 fitted with the Ornstein-Zernike function.

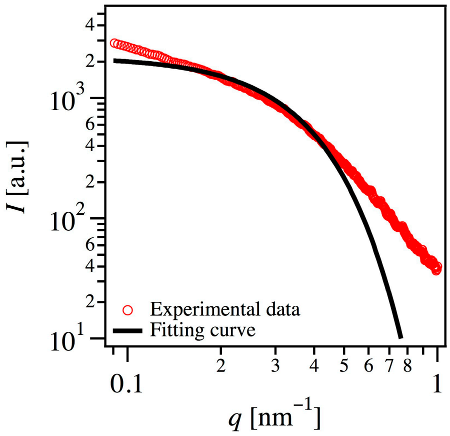

Figure S6. A circularly averaged SAXS intensity profile of the carboxylated SR gels at ϕ_a = 0.58 fitted with the Guinier function.

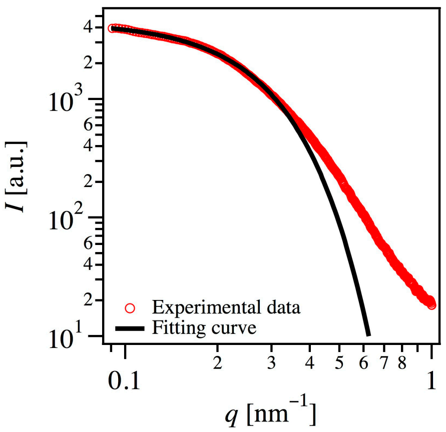

Figure S7. A circularly averaged SAXS intensity profile of the carboxylated SR gels at $\phi_a = 0.68$ fitted with the Guinier function.

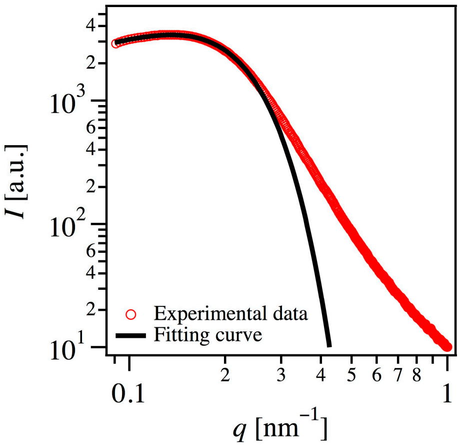

Figure S8. A circularly averaged SAXS intensity profile of the carboxylated SR gels at ϕ_a = 0.76 fitted with Equation (3).5. Swelling-Shrinking Behaviors of the SR Gels

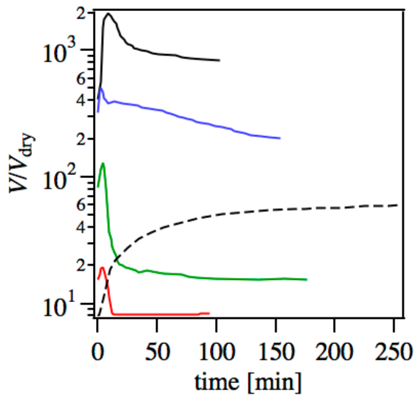

Figure S9. The degree of swelling V/V_{dry} of the carboxylated SR gels in acetone-water mixtures as a function of time when solvent was changed from $\phi_a = 0.40$ to $\phi_a = 0.30$ (dashed black line), from $\phi_a = 0.20$ to $\phi_a = 0.30$ (solid black line), from $\phi_a = 0.30$ to $\phi_a = 0.40$ (solid blue line), from $\phi_a = 0.40$ to $\phi_a = 0.50$ (solid green line), and from $\phi_a = 0.50$ to $\phi_a = 0.60$ (solid red line).

5. Vertically shifted SAXS profiles

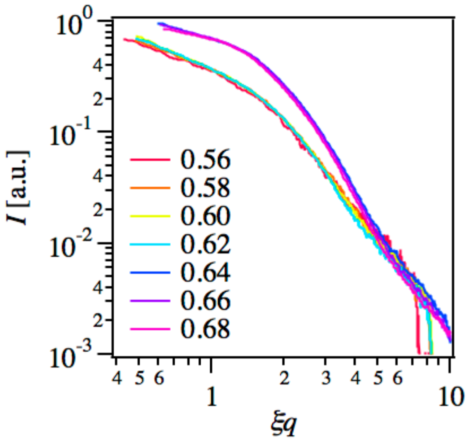

Figure S10. Vertically shifted SAXS profiles of ϕ_a = 0.56–0.68 as a function of ζq. The profiles are classified to two master curves.

© 2016 by the authors; licensee MDPI, Basel, Switzerland. This article is an open access article distributed under the terms and conditions of the Creative Commons Attribution (CC-BY) license (http://creativecommons.org/licenses/by/4.0/).
